# Efficacy and safety of Chinese herbal medicine for Coronavirus disease 2019: A protocol for systematic review and meta-analysis

**DOI:** 10.1097/MD.0000000000020157

**Published:** 2020-05-29

**Authors:** Maoyi Yang, Zhipeng Hu, Rensong Yue

**Affiliations:** Hospital of Chengdu University of Traditional Chinese Medicine, Chengdu, Sichuan Province, P.R. China.

**Keywords:** Chinese herbal medicine, Coronavirus disease 2019, protocol, systematic review and meta-analysis

## Abstract

**Background:**

Coronavirus disease 2019 (COVID-19) is a global pandemic caused by the Severe Acute Respiratory Syndrome Coronavirus-2 (SARS-CoV-2). There is no specific cure for this disease, and the clinical management mainly depends on supportive treatment. Traditional Chinese medicines (CHM) is widely used in treating COVID-19 in China.

**Methods:**

A comprehensive literature search will be conducted. Two methodological trained researchers will read the title, abstract, and full texts and independently select the qualified literature according to inclusion and exclusion criteria. After assessment of the risk of bias and data extraction, we will conduct meta-analyses for outcomes related to COVID-19. The heterogeneity of data will be investigated by Cochrane χ^2^ and *I*^*2*^ tests. Then publication bias assessment will be conducted by funnel plot analysis and Egger test.

**Results:**

The results of our research will be published in a peer-reviewed journal.

**Conclusion:**

Our study aims to systematically present the clinical evidence of CHM in the treatment of COVID-19, which will be of guiding significance for further research and clinical practice.

**OSF registration number:**

10.17605/OSF.IO/H7GMU.

## 1 Introduction

Coronavirus disease 2019 (COVID-19) is a global pandemic caused by the Severe Acute Respiratory Syndrome Coronavirus-2 (SARS-CoV-2).^[1]^ The patients of COVID-19 usually present with fever, cough, while about 23.7% of patients are accompanied by at least 1 coexisting disease.^[2–6]^ The disease is highly contagious with a R0 value about 3 to 4.^[7]^ The rapid increase in the number of patients put great pressure on the health care system. With the aggravation of the shortage of medical treatment, the mortality rate of the disease increases.^[8]^ As of 15:00 on March 31, 2020, 793,278 people were diagnosed with the disease and at least 38,545 died.

At present, there is no specific cure for this disease, and the clinical management mainly depends on supportive treatment.^[9]^ Lopinavir-Ritonavir was once thought to be a promising medicine on COVID-19, but a recently published clinical trial found that the effect was limited.^[10]^ As excessive immune response is an important reason for the progression of the patient's condition, the researchers turned their attention to immunosuppressants.^[11]^ In a small sample clinical trial, the researchers found that hydroxychloroquine could improve the state of patients’ lungs and increase the probability of the virus turning negative.^[12]^ However, the sample size of this study is small, and there may be some defects in the research design. In a case report, Remdesivir has shown good efficacy in patients with COVID-19, and larger clinical trials are currently under way.^[13]^ Therefore, it is an important and urgent task to explore new therapeutic agents.

In the fourth edition of the diagnosis and treatment guideline of COVID-19 in China, Lianhuaqingwen capsule was listed as one of the recommended medicines.^[14–16]^ In addition, traditional Chinese medicines (CHM), including Qingfei Paidu decoction, Huanglian Jiedu decoction etc., are also widely used in the adjuvant treatment of COVID-19. In terms of clinical research, many scholars have carried out clinical trials on the efficacy of CHM in treating COVID-19. However, these studies are only carried out in local areas, so they fail to provide definite evidence to prove the effectiveness of CHM. In this study, we aim to summarize the current evidence of CHM in treating COVID-19 through systematic review and meta-analysis. This study is necessary before further large-scale clinical studies being carried out. For clinicians, this study can provide some direction and guidance for clinical practice.

## 2 Methods and analysis

### 2.1 Study registration

This study has been registered at Open Science Framework (OSF, https://osf.io/) with a registration DOI: 10.17605/OSF.IO/H7GMU. This systematic review protocol is reported in accordance with the Preferred Reporting Items for Systematic Reviews and Meta-analysis Protocols (PRISMA-P) checklist.^[17]^

### 2.2 Inclusion and exclusion criteria

#### 2.2.1 Study design

Randomized controlled trials (RCTs) can provide evidence about efficacy of intervention, so they will be included in this systematic review. However, the outbreak of COVID-19 is an urgent public health event, and it is difficult to carry out RCTs, so non-randomized controlled trials will also be included in this study, although non-randomized controlled trials (non-RCTs) may be more biased than RCTs.

#### 2.2.2 Participants

Participants with a laboratory-confirmed COVID-19 diagnosis will be included in this study. There will be no limitation about kits and detection methods. Also, there will be no restriction about age, sex, and severity of disease of participants.

#### 2.2.3 Intervention

CHM in intervention group will be included. There will be no restrictions on the types, dosage forms, doses, and methods of use of CHM.

#### 2.2.4 Outcomes

Since there are no core outcome sets for COVID-19, it is difficult to predefined what outcomes will be included in our study. In general, any outcome that can reflect the condition will be included in this study.

### 2.3 Study search

Three English database including PubMed, Embase, Cochrane Library Central Register of Controlled Trials, and 4 Chinese databases including China National Knowledge Infrastructure (CNKI) database, Wanfang Data Knowledge Service Platform, the VIP information resource integration service platform (cqvip), China Biology Medicine Disc (Sino Med) will be searched from its inception to April 1, 2020 without language limitation. Preprinted website including arXiv (http://arxiv.org/), BioRxiv (https://www.biorxiv.org/), F1000 (https://f1000.com/), and PeerJ Preprints (https://peerj.com/preprints/) will also be searched to find out more unpublished papers. In addition, Chinese Clinical Trial Registry (ChiCTR) and ClinicalTrials.gov will also be searched to find out ongoing research.

A search strategy of the combination of controlled vocabulary and text words will be adopted. Boolean operators will be used to concatenate search terms. This work will be conducted by 2 authors (ZH and MY) independently. The search strategy of PubMed is presented in Table 1.

**Table 1:**
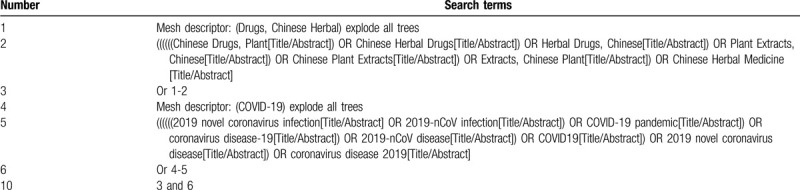
Example of PubMed search strategy.

### 2.4 Study selection

**Figure 1:**
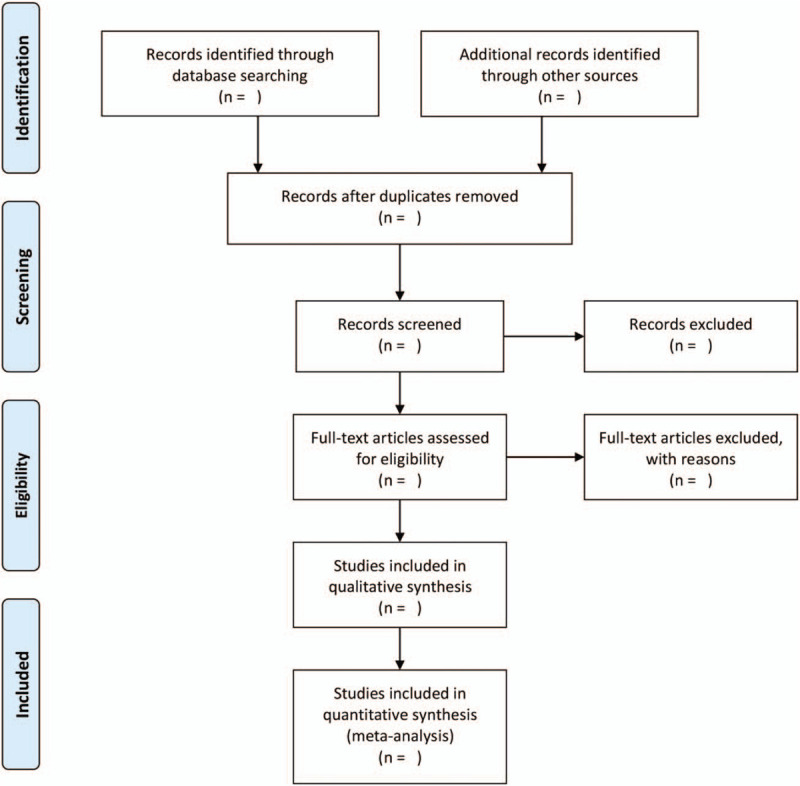
Flow chart of study selection.

EndNote X9 will be used by 2 researchers (ZH and MY) to screen the citations independently according to the predefined inclusion and exclusion criteria. Discrepancies between 2 authors will be solved by discussion with a third author (RY). A research flow chart will be drawn to show the whole process of research selection (Fig. 1).

### 2.5 Data extraction

Data extraction will be conducted by 2 independent authors (MY and ZH) according to a prespecified form and checked by a third author (RY). The following data will be extracted: the first author's name, publication time, country, article title, article type, interventions in experimental and control group, course of treatment, severity of disease, number of patients in each group, ages and sex of patients, outcomes and adverse effect.

### 2.6 Risk of bias assessment

Different risk of bias assessment tools will be used according to different types of research. The risk of bias of RCTs will be conducted using version 2 of the Cochrane risk-of-bias tool for randomized trials (RoB2).^[18]^ The Risk of Bias In Non-randomized Studies of Interventions (ROBINS-I) tool will be used to assess the risk of bias of non-RCTs according to Cochrane Handbook.^[19]^

### 2.7 Data analysis

Data analysis will be conducted using Stata 14.0, StataCorp, Texas, USA. The effect measure of binary variable will be expressed as risk ratio (RR) or odds ratio (OR) and 95% confidence interval (CI). For continuous variables, 95% CI and mean difference (MD) or standardized mean difference (SMD) will be used. The number needed to treat (NNT) will be calculated for the interpretation of results. Cochrane *χ*^2^ and *I*^*2*^ tests will be conducted to assess the heterogeneity analysis between studies. When *P* < .05 and *I*^*2*^ > 50%, a random effect model will be used. When *P* > .05 and *I*^2^ < 50%, then a fixed effect model will be used to calculate the effect size. The results of RCTs and non-RCTs will be analyzed and presented independently. Subgroup analysis will be conducted to explore the subgroup effects and investigate the source of heterogeneity. If there is a substantial heterogeneity and quantitative synthesis is not appropriate, the results will be presented in the form of tables and figures.

Publication bias and small-study effects will be evaluated by funnel plot and statistically investigated by Egger test with a *P* value boundary of .05.^[20]^

### 2.8 Ethics and dissemination

Meta-analysis is an analysis of previous research data and does not require ethical approval. The results of this study will be published in peer-reviewed journals.

## 3 Discussion

COVID is a global epidemic that has so far caused >700,000 confirmed cases and 30,000 deaths. At present, there is no effective treatment. Due to the long cycle and difficulties of new drug research and development, mining existing drugs has become the focus of research. Since there are some difficulties in carrying out clinical trials, this study will include both RCT and non-RCTs. Non-RCTs may introduce bias in the course of research, but it can provide evidence more conveniently. This means that we need to be more careful when interpreting non-RCT results. This research will comprehensively present the existing evidence of CHM in treating COVID-19, which will be of guiding significance for further research.

### 3.1 Amendments

If any modification is required, we will update our protocol to include any changes in the entire research process.

## Author contributions

**Conceptualization:** Maoyi Yang, Zhipeng Hu, Rensong Yue.

**Data curation:** Maoyi Yang, Zhipeng Hu.

**Formal analysis:** Maoyi Yang, Zhipeng Hu.

**Investigation:** Maoyi Yang, Zhipeng Hu.

**Methodology:** Maoyi Yang, Zhipeng Hu.

**Project administration:** Rensong Yue.

**Software:** Maoyi Yang, Zhipeng Hu.

**Visualization:** Maoyi Yang, Zhipeng Hu.

**Writing – original draft:** Maoyi Yang.

**Writing – review and editing:** Rensong Yue.
